# A Novel Column-Switching Method Coupled with Supercritical Fluid Chromatography for Online Analysis of Bisphenol A Diglycidyl Ether and Its Derivatives in Canned Beverages

**DOI:** 10.3390/molecules30071565

**Published:** 2025-03-31

**Authors:** Chaoyan Lou, Shaojie Pan, Kaidi Zhang, Xiaolin Yu, Kai Zhang, Yan Zhu

**Affiliations:** 1 College of Quality and Standardization, China Jiliang University, Hangzhou 310018, China; 18868730104@163.com (S.P.);; 2 Department of Chemistry, Zhejiang University, Hangzhou 310028, China; 3 Ningbo Key Laboratory of Agricultural Germplasm Resources Mining and Environmental Regulation, College of Science and Technology, Ningbo University, Ningbo 315300, China; zhangkai1@nbu.edu.cn

**Keywords:** bisphenol A diglycidyl ether (BADGE), BADGE derivatives (BADGEs), column switching, supercritical fluid chromatography, canned beverages, emerging pollutants

## Abstract

Bisphenol A diglycidyl ether (BADGE) and its related derivatives (BADGEs for short) are reactive epoxides condensed from bisphenol A (BPA) and epichlorohydrin. Nowadays, they are heavily used as additives in the production process of food and beverage contacting materials. However, BADGEs are considered as emerging organic pollutants due to their high toxicity including cytotoxicity, mutagenicity, and genotoxicity. In this work, an online analytical method integrated column-switching technique with supercritical fluid chromatography (SFC) was proposed for the simultaneous determination of bisphenol A diglycidyl ether and its derivatives. In this process, a homemade column was utilized in the first dimension of the column-switching SFC system to preconcentrate the analytes as well as eliminate interferences online. Under the optimal conditions, the obtained calibration curves for BADGEs showed good linearity ranging from 0.02 μg/mL to 10.00 μg/mL, while the values of LOD and LOQ were in the range of 0.0024–0.0035 μg/mL and 0.0080–0.0116 μg/mL, respectively. The optimized method exhibited a good recovery ranging from 85.6% to 105.5% with relative standard deviations less than 11.8%. The developed method provides an eco-friendly and effective way for the rapid and automated analysis of BADGEs at trace levels in canned beverages and can be applied to the high-throughput analysis of other similar matrices.

## 1. Introduction

**Figure 1:**
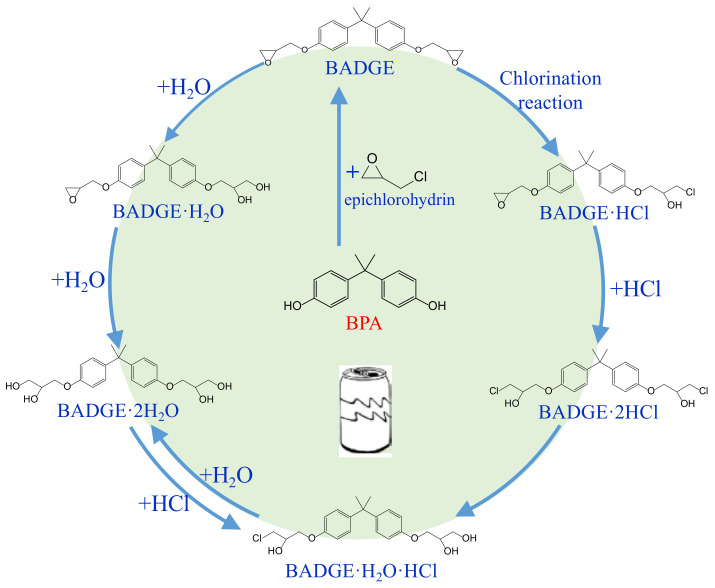
The transformation path of BADGE and its derivative molecules.

Bisphenol A diglycidyl ether (BADGE) is a synthetic compound generated through a condensation reaction between epichlorohydrin and bisphenol A (BPA). BADGE is widely used as a precursor in the production of a wide range of polymerization products [1]. It is consumed as a starting material for producing epoxy resins as well as an additive for the elimination of surplus hydrochloric acid in polyvinyl chloride (PVC) organosols. These polymerization products are frequently applied as protective linings to prevent corrosion in cans and food containers [2]. During the polymerization process, BADGE in contact with aqueous and acidic foods results in the formation of its hydrolysis derivatives called bisphenol A (2,3-dihydroxypropyl) glycidyl ether (BADGE·H_2_O) and bisphenol A bis(2,3-dihydroxypropyl) ether (BADGE·2H_2_O). Furthermore, BADGE can also be transformed into its chlorinated derivative products through the major chlorination reaction, which are called bisphenol A (3-chloro-2-hydroxypropyl) glycidyl ether (BADGE·HCl), bisphenol A bis(3-chloro-2-hydroxypropyl) ether (BADGE·2HCl) and bisphenol A (3-chloro-2-hydroxypropyl)(2,3-dihydroxypropyl ether) (BADGE·H_2_O·HCl) [3]. The formation procedures of BADGE and its derivative molecules are presented in Figure 1.

BADGE and its derivatives (presented in Table 1) can easily migrate from food packaging materials into foods due to their high reactivity, which may cause contamination in the food matrix and finally endanger human health [4,5]. In order to evaluate the potential risks on the consumers, there are more and more studies focusing on the toxicity of BADGE and its hydrated and chlorinated derivatives. Several reports have indicated that BADGE and its derivatives have rather specific mutagenic, genotoxic and cytotoxic actions in some tissues and cell models [6,7,8,9]. The presence of this family of compounds in foodstuffs has received more and more attention due to their adverse effects. To avoid the health risks from these emerging pollutants and to ensure the quality of beverages, several institutions have prohibited or restricted the use of BADGEs in food contact materials. For example, European Union (UN) legislation establishes that the specific migration limits (SML) shall not exceed 9 mg/kg for the sum of BADGE and its hydrolysis derivatives and 1 mg/kg for the chlorohydroxy derivatives [10].

In this perspective, a study on the content of BADGE and its derivatives in foodstuffs is of great significance to public health and regulation guidance. Canned beverages are representative food samples with potential BADGE contamination risks, since liquids can accelerate the migration of BADGEs from the can coating material into the foodstuff [11]. However, due to the low concentrations of BADGEs and the high contents of beverage ingredients, publications and research on the problem of determining BADGEs in beverage matrices are insufficiently available. To overcome this problem, a sensitive and selective analytical method to monitor the occurrence and content of BADGEs in such foodstuffs needs to be established urgently.

Since the complexity of matrix effects continues being the major concern for the analysis of this family of compounds in canned foods, appropriate sample preparation needs to be further studied. Conventional sample preparation techniques including liquid-liquid microextraction (LLME) [12], solid phase extraction (SPE) [13,14,15], QuEChERS [16], and solid phase microextraction (SPME) [17] have been studied to eliminate the influence of the matrix. Each of these techniques has its own merits, but these procedures always require multiple steps and use high volumes of organic solvents. Moreover, manual operation in these procedures is not always satisfactory due to its inevitable artificial errors. Hence, such offline pretreatment methods are not the first choice to meet the requirements of high-throughput analysis. To address the problem, the online sample preparation technique has turned out to be a better solution due to its automation [18]. The column switching technique is an online connection mode in which sample preparation steps are integrated with a chromatographic separation procedure to reduce the potential contamination introduced by sample transfer and achieve selective enrichment directly in the routine process [18,19,20].

Furthermore, the development of the routine analysis of trace BADGEs not only requires an indispensable preparation process, but also an effective instrumental analysis method. As comprehensively reviewed, BADGE and its derivatives are regularly analyzed by high performance liquid chromatography (HPLC) [21,22], liquid chromatography coupled mass spectrometry (LC-MS) [23,24], gas chromatography coupled mass spectrometry (GC-MS) [25], or immunochromatographic strip assay [6]. These methods require a long analysis time (>30 min) that cannot meet the requirements of high-throughput analysis. Recently, the state-of-the-art approach based on supercritical fluid chromatography (SFC) has emerged as a new methodology for analyzing BADGEs in the food matrix, which performs with greater chromatographic efficiency and a shorter analysis time [26,27,28]. Additionally, SFC exhibits several impressive properties such as low mass transfer resistance, high density, low viscosity and environmental friendliness. It is unambiguous that the detection of BADGEs with SFC in complicated matrices still needs to be further studied, since the analysis of BADGEs by SFC has rarely been reported in previous studies.

In the present study, the column switching sample treatment process and supercritical fluid chromatographic separating operation were integrated into an online system to simultaneously analyze BADGE and its derivatives, which substantially reduced the analytical time and decreased the artificial operation errors. Through the design of the column switching methodology, the system was able to automatically remove matrix interferences and achieve an online concentration of BADGEs. After the optimization of conditions, this efficient, rapid, and environmentally friendly method was validated and applied to identify BADGEs in canned beverages.

## 2. Results and Discussion

### 2.1. Optimization of Online Column Switching Procedure

The column switching-SFC system was composed of SFC instruments and an external column switching module to implement the online preparation of samples. There were three steps involved in the determination of BADGEs by the column switching-SFC system: (A) system regeneration and sampling; (B) target molecule enrichment and matrix elimination; and (C) analysis and detection of BADGEs in supercritical fluid chromatography.

**Figure 2:**
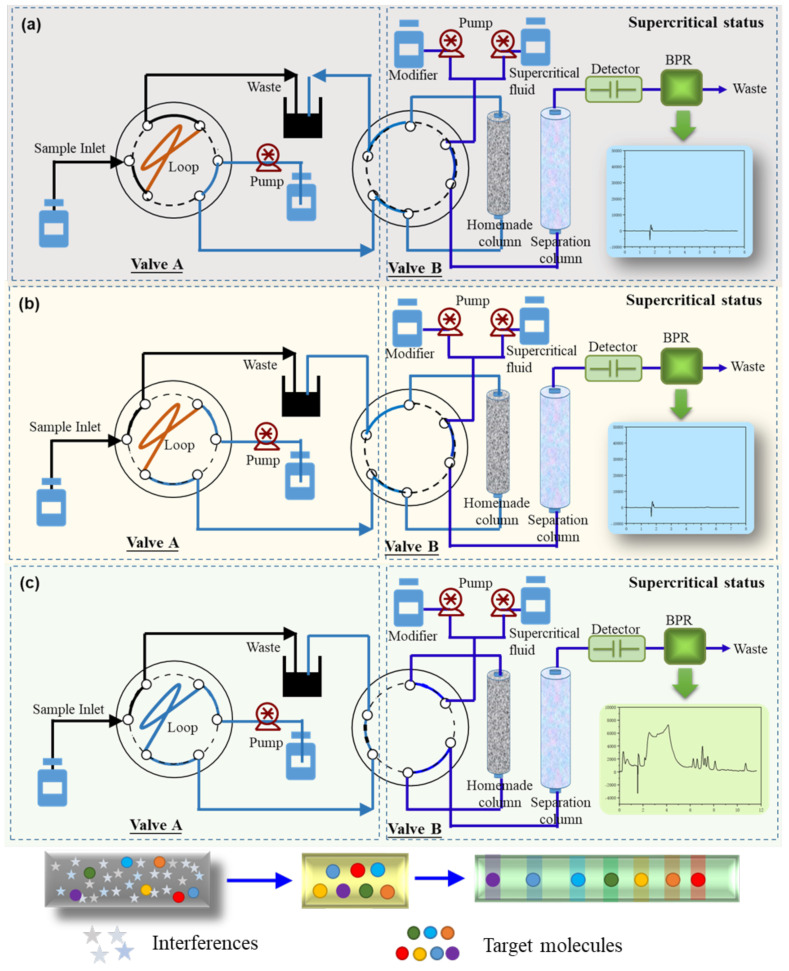
Configuration diagram of the online column switching-SFC system. (**a**) sample loading as well as system regeneration; (**b**) target molecule enrichment and matrix elimination; and (**c**) analysis and detection of BADGEs in supercritical fluid chromatography.

The configuration diagram of the column switching via a simple single pump is illustrated in Figure 2. As shown in Figure 2a, in the first step, 1 mL of sample is injected into the loop in Valve A at the state of load position. Then, Valve A is switched to the inject position, eluting the sample into the homemade column (Figure 2b). At this step, the analytes are retained and enriched on the homemade column. In the meantime, impurities that have no retention behavior on the homemade column are directly washed out by the external pump. Subsequently Valve B is switched to the inject position, connecting the homemade column to the analytical column so that the analytes are delivered into the separation system (Figure 2c), where detection is finally achieved. In the meantime, the valves are restored to the initial state to regenerate for the next batch.

**Figure 3:**
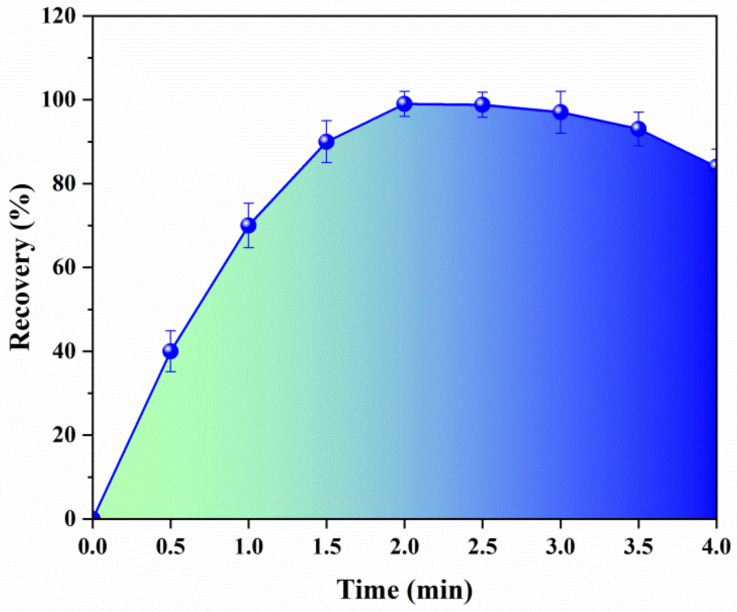
Influence of switching time on the extraction recovery.

Optimization of the column switching system was conducted. In the column switching protocol, a homemade column according to our previous work [29] was adopted instead of a commercial column to enrich BADGE and its derivatives. The column was packed with poly(GMA-DVB) microspheres functionalized with octadecylamine (20 μm, 4.6 mm × 150 mm) according to the two-staged swelling and polymerization method. For an online preparation procedure, the switching time should be programmed precisely since it determines whether the sample can be fully and selectively washed into the analytical column or not. To achieve the highest extraction yields for BADGE and its derivatives, different switching times were investigated and optimized in this step. Other parameters including the sample volume, eluent solvent for homemade column, and flow rate remained constant. Figure 3 shows the dependence of the extraction yields upon the switching time, ranging from 0 min to 4 min under the mixed eluent solvent of H_2_O-CH_3_OH (1:9, *v*/*v*). The extraction yields of all BADGEs increased from 0 min to 2 min and reached the maximum at around 2 min. With the switching time of 2 min, the extraction yields of all BADGEs could be sustained at a high level (not less than 90%), and with the lasting switching time, the yields of the BADGEs decreased gradually. Hence, the switching time in the proposed research was finally set at 2 min. Table 2 depicts the detailed operation instructions for the complete procedure.

### 2.2. Optimization of SFC Conditions

In this study, SFC conditions including the stationary phase and mobile phase were investigated in order to improve the separation behavior of seven BADGEs. BADGE and its derivatives are structurally close molecules that contain two propane-bridged aromatic rings with each aromatic ring bearing an epoxy group or an additional substituent. In this research, we focused on the seven most prevalent BADGEs, as summarized in Table 1. Given the similarity of the BADGE structures, the isolation of seven BADGEs by chromatography would be difficult to achieve.

Chromatography separation is related to the specific interactions formed between the BADGEs and the stationary phase, thus the screening experiment of the appropriate stationary phase is crucial to the chromatography separation in SFC. According to the unique property of supercritical fluid, either a normal phase column or reversed phase column can be involved in the supercritical fluid chromatography system. Considering the weak polarity of BADGE and its derivatives, reversed phase columns were inclined to attain the appropriate separations and desirable retention. In preliminary experiments, a column screening test was conducted with three different types of commercial stationary phases including an Ultimate XB-C18 (5 μm, 4.6 mm × 250 mm), Eclipse XDB-C8 (5 μm, 4.6 mm × 250 mm), and Ultimate XB-C4 (5 μm, 4.6 mm × 250 mm). The three columns were tested to separate seven BADGEs with concentrations of 0.1 μg/mL under the same mobile phase condition and other equal operating parameters (temperature, backpressure and flow rate). The three columns exhibited quite different behaviors on the separation of BADGEs. The column with longer carbon chains showed better chromatographic performance. The Ultimate XB-C4 column grafted with butyl groups showed a weak retention capacity and was unable to separate the analytes. The Eclipse XDB-C8 column with the octyl group demonstrated a better retention behavior than the Ultimate XB-C4 column; however, it is worth noting that the separation performance of the seven BADGEs was disturbed by peak tailing. It was observed that the Ultimate XB-C18 column with a longer carbon chain was more effective in separating BADGEs than the Eclipse XDB-C8 and Ultimate XB-C4. Complete baseline separation and good symmetrical peak shape were achieved on the Ultimate XB-C18 column. Hence, the Ultimate XB-C18 column was selected for subsequent investigations.

The mobile phase is another main parameter that affects the separation of BADGEs in SFC. The influence of the mobile phase conditions (the composition of mobile phase and the percentage of modifier) on the retention and selectivity were investigated and discussed. In SFC, supercritical CO_2_ is usually used as the major component of the mobile phase; however, pure supercritical CO_2_ is a nonpolar fluid that has poor elution for BADGEs. Thus, a polar co-solvent is always added as a modifier to regulate the solvation power. In this experiment, different modifiers including methanol, ethanol, isopropanol, and acetonitrile were mixed with supercritical CO_2_. The results demonstrated that methanol was the optimal choice due to its better polarity and lower viscosity. Furthermore, as shown in Table 1, the polarity of all BADGEs was quite weak. This implies that a small amount of modifier would be sufficient to enhance the separation property. Different quantities of methanol ranging from 1% to 10% were evaluated. When the proportion of methanol remained at 4%, the BADGEs were well-separated in the SFC system. Under this condition, the peak shapes of the BADGEs were quite symmetrical and narrow and the analysis time was short. However, when the proportion of methanol was set at more than 5%, all the BADGEs were washed out immediately and fused together. Given the results, a low quantity of methanol (4%) was added into the supercritical fluid.

Except for the mobile phase, other parameters including the flow rate, temperature, and back pressure were also investigated to improve the efficiency of separations. These parameters can predominantly affect the density and viscosity of supercritical fluids. In SFC, the effects of temperature on retention behavior are much more complicated than that in HPLC. Temperature not only influences the mass transfer rate of the target molecules, but also impacts the density and viscosity of the supercritical fluid. After testing the separation at temperatures from 35 °C to 45 °C, 40 °C was chosen as the optimal temperature where complete separation was accepted within 12 min. Moreover, due to the high diffusivity and low viscosity of the mobile phase, a high flow rate of 2.0 mL/min was occupied in the SFC system, while a back pressure of 10 MPa was programmed to maintain the supercritical status of CO_2_.

**Figure 4:**
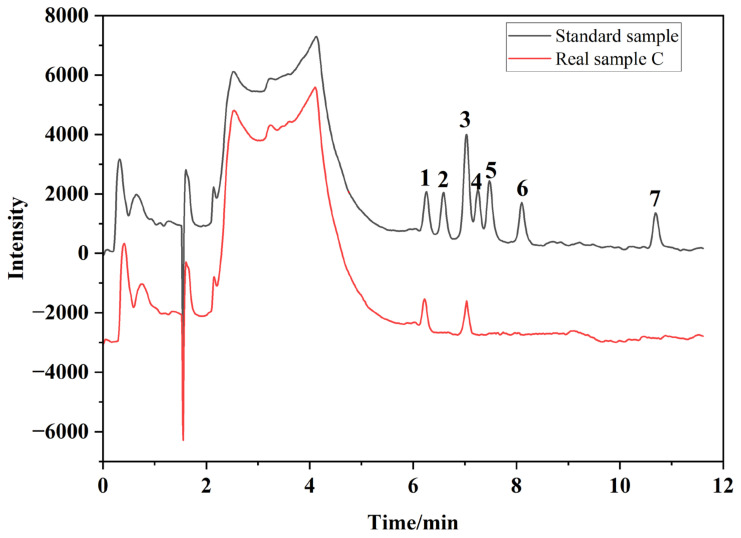
Chromatogram obtained of a mixed BADGE standard sample (0.10 μg/mL) and a real sample. The peaks represent: (1) BADGE·2H_2_O, (2) BADGE·H_2_O·HCl, (3) BPA, (4) BADGE·H_2_O, (5) BADGE·2HCl, (6) BADGE·HCl, and (7) BADGE.

Given the above, the operation conditions of SFC for analyzing BADGE and its derivatives were as follows: an Ultimate XB-C18 column (i.d. 5 μm, 4.6 mm × 250 mm) was used as the stationary phase with temperature maintained at 40 °C. The mobile phase was supercritical carbon dioxide mixed with methanol to regulate the polarity of the eluent. The optimum mobile phase was maintained at 4% modifier at a flow velocity of 2.0 mL/min. The back pressure was maintained at 10 MPa. The UV wavelength was set at 230 nm. Under these conditions, the obtained chromatogram of the results, as presented in Figure 4, appeared to be accurate and it was possible to eliminate the matrix effects.

### 2.3. Method Validation

A validation study was performed including linearity, recovery, precision, LOD, and LOQ to ensure the accuracy and reliability of the proposed method for routine analysis. In order to assess the linearity, six-point matrix matched calibration curves (0.02 μg/mL to 10.00 μg/mL) were achieved by using a blank matrix extract spiked with a certain amount of the target compounds. As shown in Table 3, the obtained calibration curves were linear in the scale of 0.02 μg/mL to 10.00 μg/mL, with the correlation coefficients above 0.9985. The LODs were calculated in the range of 0.0024–0.0035 μg/mL, while the LOQs were 0.0080–0.0116 μg/mL based on 3 and 10 times the signal-to-noise (S/N) ratio, respectively.

Furthermore, the precision and accuracy of the developed method were also evaluated though spiked recovery testing using spiked blank beverages at three concentration levels (0.05 μg/mL, 0.50 μg/mL, 5.0 μg/mL) containing six parallel experiments (*n* = 6). The results in Table 4 indicate that the spiked recoveries of all seven compounds ranged from 85.6% to 105.5%, while the RSD of all batches in this testing was below 11.8%.

### 2.4. Method Application in Real Samples

The validated column switching-SFC method was applied in the determination of trace BADGEs in canned beverages under the optimum conditions. The samples were collected from local supermarkets randomly. The contents of BADGEs in the real samples were calculated with the external standard method. Among all of the samples, BADGE·2H_2_O was found in three samples, with its concentrated contents ranging from 0.036 to 0.063 μg/mL, while BADGE and another hydrolysis derivative BADGE·H_2_O were found to be 0.022 μg/mL in sample D and 0.021 μg/mL in sample E, respectively. Additionally, BADGE·H_2_O·HCl was only detected in one sample with the content of 0.024 μg/mL, indicating that chlorohydroxy derivatives of BADGE are less prevalent in foodstuffs. The results of all the samples are summarized in Table 5. In general, although the contents of BADGEs in the collected samples did not exceed the specific migration limits stipulated by European Union, their chronic low-dose exposure to foodstuffs remains a concern, and their possible cumulative effects should not be overlooked.

### 2.5. Comparison with Other Methods

To highlight the advantages of the proposed method, a comparison of the column switching integrated with SFC method with other methods reported in the literature is summarized in Table 6. The total operating time of the proposed method is distinctly shorter than the previously published methods, indicating that this column switching-SFC method is applicable for routine analysis. Moreover, published methods always require laborious offline operations with inevitable manual errors, while this method adopts an online pattern to eliminate such manual errors. Notably, the methodologies of BADGE analysis reported in the literature are mostly based on the liquid chromatography technique, whereas supercritical fluid chromatography has seldom been considered in the identification of BADGEs. Notwithstanding the high throughput and rapid analysis, the sensitivity of the current method is not as good as HPLC-MS. This can be attributed to the detector used in the SFC system and can be further improved in future work by equipping it with mass spectrometry. On the whole, the proposed method is a rapid, ecofriendly, sensitive, and robust approach, and has emerged as good alternative for the determination of BADGEs in canned beverages.

## 3. Materials and Methods

### 3.1. Reagents and Materials

Bisphenol A diglycidyl ether (BADGE; 98.0%) and its derivatives, BADGE monohydrate (BADGE·H_2_O; ≥95.0%), BADGE dihydrate (BADGE·2H_2_O; ≥97.0%), BADGE hydrochloride (BADGE·HCl; ≥90.0%), BADGE dihydrochloride (BADGE·2HCl; ≥97.0%), and BADGE chlorohydroxy (BADGE·H_2_O·HCl; ≥95.0%) of analytical grade were purchased from Sigma-Aldrich (St. Louis, MO, USA). Acetonitrile, methanol, ethanol, and isopropanol of HPLC grade were supplied by the Tedia Company (Fairfield, CT, USA). SFC-grade carbon dioxide (purity ≥99.9999%) stored in a high-press gas cylinder was provided from Hangzhou Jingong Special Gas Co. Ltd. (Hangzhou, China). Ultrapure water (18.2 MΩ·cm) was obtained from a Milli-Q purification system (Millipore, MA, USA).

Poly(GMA-DVB) microspheres functionalized with octadecylamine were synthesized by the two-staged swelling and polymerization method, referring to previous work. These were further packed with a homemade column (4.6 mm × 150 mm).

### 3.2. Preparation of Standard Solutions and Canned Beverage Samples

Stock solutions of individual bisphenols were prepared by dissolving 50 mg of each BADGE standard sample into 50 mL of methanol and stored at −4 °C. Working standard solutions were prepared daily by diluting the stock solutions with liquid food simulants to the appropriate concentration ranges. Canned beverage samples of different brands were collected randomly from local supermarkets (Hangzhou, China). Plastic apparatus was forbidden across the whole procedure to avoid possible contamination. Sample solutions were filtered through a 0.22 μm filter before injection. The filters were pre-washed with a methanol/water (50:50, *v*/*v*) mixed solution to eliminate potential additive contamination.

### 3.3. Instrumentation

Chromatographic separation was conducted on a Shimadzu Nexera UC SFC system with UV detector (Shimadzu, Japan). This supercritical fluid chromatography system was configured with a supercritical fluid delivery pump and a quaternary liquid pump, respectively. Other modules including a column oven, online degasser, back pressure regulator, and ultraviolet detector were also added to the SFC system to maintain the supercritical status of the whole system.

### 3.4. Fabrication of Online Column Switching System

The online system was achieved through a column-switching protocol. The major accessories of the online protocol included two six-port valves, a liquid infusion pump, and a homemade column. The separation of BADGEs was conducted on a Shimadzu Nexera UC SFC system as mentioned in Section 3.3. A supercritical fluid delivery pump was used to carry supercritical CO_2_ while the quaternary liquid pump was used to carry a polarizing modifier agent. The external pump delivered the eluting agent composed of aqueous solution:methanol (10:90, *v*/*v*). The whole assembly of the SFC is plotted in Figure 2. As shown in Figure 2, the procedure involved three steps to accomplish the online analysis of BADGE derivatives.

## 4. Conclusions

In recent years, the development of advanced analytical methodologies for the rapid and precise determination of emerging contaminants such as BADGEs has been a pivotal challenge to human health and environmental sustainability. In this research, a column switching-SFC method was established for the online simultaneous determination of seven BADGEs in canned beverages. Under the optimum conditions, the BADGEs could be separated simultaneously within 12 min. The behavior of the proposed method was validated in terms of linearity, sensitivity, precision, and accuracy. The results indicate that this method exhibits good sensitivity, desirable rapidity, rugged reliability, and high efficiency, which is suitable for high-throughput routine analysis. Moreover, the reported method using SFC was much cleaner and greener, as few organic solvents were consumed, aligning with green chemistry principles. Depending on the above-mentioned superiorities, this approach can be a promising alternative for regulatory applications, as its sensitivity meets the SML requirements, and its automation enables high-throughput screening. For future food safety monitoring, this method can be integrated with high resolution mass spectrometry (HRMS) to expand its scope to other even more complicated matrices such as lipid-rich food products and biological samples.

## Figures and Tables

**Table 1:** The information of BADGE and BADGE-related molecules studied in the present study.

Compound	Formula	CAS No.	Molecular Weight	Dipole Moment ^a^	Chemical Structure
BPA	C_15_H_16_O_2_	80-05-7	228.29	2.15	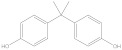
BADGE	C_21_H_24_O_4_	1675-54-3	340.41	2.58	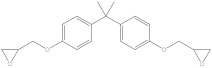
BADGE·H_2_O	C_21_H_26_O_5_	76002-91-0	358.43	4.24	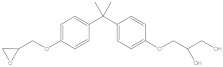
BADGE·2H_2_O	C_21_H_28_O_6_	5581-32-8	376.44	2.59	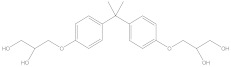
BADGE·HCl	C_21_H_25_ClO_4_	13836-48-1	376.87	2.76	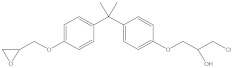
BADGE·2HCl	C_21_H_26_Cl_2_O_4_	4809-35-2	413.33	1.21	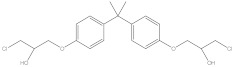
BADGE·H_2_O·HCl	C_21_H_27_ClO_5_	227947-06-0	394.89	3.68	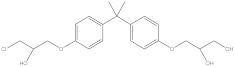

^a^ The values of dipole moments were calculated in Chem 3D software (Version 13.0) with integrated Gaussian functionality.

**Table 2:** Programmed procedure for the online column switching-SFC system.

Procedure	Description	Time/min	Valve A ^a^	Valve B ^b^
Step A	Sampling/regeneration	Initial State/12.0–15.0	Load	Load
Step B	Enrichment	0.0–2.0	Inject	Load
Step C	Analysis and Detection	2.0–12.0	Inject	Inject

^a^ Eluent of the homemade column was a mixture of H_2_O and methanol (1:9, *v*/*v*) at a flow rate of 1.0 mL/min. ^b^ Eluent of the SFC column was supercritical state CO_2_ with methanol as the modifier.

**Table 3:** Linear ranges, correlation coefficients, LOD, and LOQ of seven BADGEs.

BADGEs	Linear Range (μg/mL)	Calibration Curve	Correlation Coefficient (R^2^)	LOD (μg/mL)	LOQ (μg/mL)
BADGE·2H_2_O	0.02–10.00	Y = 26201.5X + 67.4	0.9990	0.0031	0.0102
BADGE·H_2_O·HCl	0.02–10.00	Y = 27965.3X + 84.9	0.9992	0.0030	0.0101
BPA	0.02–10.00	Y = 39716.2X − 364.2	0.9987	0.0024	0.0080
BADGE·H_2_O	0.02–10.00	Y = 24496.8X − 210.3	0.9985	0.0035	0.0116
BADGE·2HCl	0.02–10.00	Y = 28567.9X − 251.5	0.9988	0.0030	0.0099
BADGE·HCl	0.02–10.00	Y = 27186.4X + 89.7	0.9995	0.0031	0.0104
BADGE	0.02–10.00	Y = 26050.2X + 85.1	0.9997	0.0033	0.0109

**Table 4:** Recoveries and precision in the blank canned beverage samples (*n* = 6).

BADGEs	Spiked Level (μg/mL)	Average Recovery (%)	Intra-Day RSD (%)	Inter-Day RSD (%)
BADGE·2H_2_O	0.05	95.6	4.3	6.1
	0.50	93.8	5.3	7.0
	5.0	94.2	5.8	8.7
BADGE·H_2_O·HCl	0.05	101.8	8.3	5.2
	0.50	94.9	5.5	7.7
	5.0	97.5	5.9	8.5
BPA	0.05	95.9	7.6	8.2
	0.50	90.5	11.6	10.4
	5.0	91.7	11.5	10.1
BADGE·H_2_O	0.05	94.8	9.8	9.5
	0.50	85.6	9.2	10.3
	5.0	86.1	11.0	11.8
BADGE·2HCl	0.05	89.0	10.3	10.4
	0.50	91.7	4.9	8.9
	5.0	88.9	11.5	10.9
BADGE·HCl	0.05	102.1	5.6	6.2
	0.50	91.6	4.5	7.8
	5.0	105.5	6.0	8.3
BADGE	0.05	96.2	2.9	4.8
	0.50	91.9	4.2	5.5
	5.0	103.6	5.1	7.6

**Table 5:** Contents of BADGEs in canned beverages by the online-SFC method (*n* = 3).

Target Molecules	Content (μg/mL)						
	Sample A	Sample B	Sample C	Sample D	Sample E	Sample F	Sample G
BADGE·2H_2_O	ND ^a^	0.063 ± 0.005	0.051 ± 0.004	ND	0.036 ± 0.003	ND	ND
BADGE·H_2_O·HCl	0.024 ± 0.002	ND	ND	ND	ND	ND	ND
BPA	ND	ND	0.038 ± 0.003	ND	ND	ND	ND
BADGE·H_2_O	ND	ND	ND	ND	0.021 ± 0.001	ND	ND
BADGE·2HCl	ND	ND	ND	ND	ND	ND	ND
BADGE·HCl	ND	ND	ND	ND	ND	ND	ND
BADGE	ND	ND	ND	0.022 ± 0.001	ND	ND	ND

^a^ ND: Not detected (<LOQ).

**Table 6:** Comparison of the proposed approach with the major approaches reported in the literature.

Method	Target Analytes	Matrix	Time	Operation Mode	Sensitivity (LOQ)	Recovery	Reference
Online with SFC	6BADGEs+BPA	Canned beverages	15 min	Online	8.0–11.6 ng/mL	89.0–108.3%	Proposed method
dSPE-SPE-HPLC-FLD	4BADGEs+3BPs	Human breast milk	>3 h	Offline	171.89–235.11 ng/mL	56.8–88.5%	[15]
DLLME-HPLC-FLD	3BADGEs+4BPs	Human breast milk	>20 min	Offline	1.4 ng/mL–6.3 ng/mL	67–110%	[22]
SPE-HPLC-FLD	1BADGEs+2BPs	Human urine	>20 min	Offline	11.42–22.35 ng/mL	73.7–87.0%	[30]
HPLC-MS	8BADGEs+13BPs	Biological fluids	>30 min	Offline	0.019–0.81 ng/mL	70–114%	[23]
GC-MS	7BADGEs+6BPs	Food cans	>20 min	Offline	/	/	[25]
Immunochromatographic strip assay	4BADGEs	Canned foods	200 min	Offline	0.97 ng/mL (LOD)	79.86–93.81%	[6]

## Data Availability

The original contributions presented in this study are included in the article. Further inquiries can be directed to the corresponding author(s).
